# Surgeon General's bone health project: translation of research to mitigate injury risk in Royal Marines recruits

**DOI:** 10.1186/1471-2474-16-S1-S15

**Published:** 2015-12-01

**Authors:** JL Fallowfield, T Davey, SA Lanham-New, AJ Allsopp

**Affiliations:** 1 Environmental Medicine and Sciences Division, Institute of Naval Medicine, Alverstoke, Hampshire, PO12 2DL, UK; 2 Nutritional Sciences Department, Faculty of Health and Medical Sciences, University of Surrey, Guildford, Surrey, GU2 7XH, UK

## 

Overuse injury, including stress fracture (SF), in military training results in significant economic losses and individual discomfort. The 32-week Royal Marine (RM) training programme is recognised worldwide as the most arduous initial training programme. Research has failed to fully identify bone density and structural differences between SF personnel and controls due to inadequate adjustment for confounding factors and poor sample size. Moreover, whilst associations have been reported between serum 25-hydroxyvitamin-D (25(OH)D) and SF risk, the threshold for this effect remains unclear. This programme determined if 25(OH)D concentrations were associated with SF risk during military training, and investigated physical and bone differences between matched (age, body size and aerobic fitness) injured and uninjured recruits, to inform future policy and practice.

RM recruits (n=1090; males aged 16-32 y) were followed through training, where 78 recruits (7.2%) suffered 92 SF in total. Anthropometric measures and aerobic fitness were assessed at week-1. Lumbar spine (LS), femoral neck (FN) and whole body (WB) Bone mineral density (BMD) (Dual X-ray Absorptiometry), and tibial bone parameters (peripheral Quantitative Computer Tomography), were measured in matched (injured vs. uninjured) pairs. Venous blood samples drawn at weeks 1, 15 and 32, were analysed for serum C-terminal peptide concentration (as a marker of bone resorption), 25(OH)D and PTH.

Age (<18 y; >27 y), body mass (<65 kg) and fitness were associated with injury risk (P<0.05). Recruits with a baseline 25(OH)D concentration <50 nmol.L^-1^ had a higher SF incidence than recruits with 25(OH)D concentration >50 nmol.L^-1^ (χ^2^_(1)_=3.564, *p*=0.042; odds ratio 1.6 (95% CI 1.0-2.6)). There were weak inverse correlations between week-15 25(OH)D and PTH concentrations (r=-0.209, *p*<0.001) and week-32 (r=-0.214, *p*<0.001), but not at baseline. BMD at the LS, WB and FN were lower in SF compared with uninjured recruits (ANCOVA, P<0.001). Structural differences between SF and uninjured recruits were evident in all slices of the tibia, but most prominently at the 38% slice. There was a negative correlation between bone cross-sectional area and BMD at the 38% tibial slice. There was no difference in CTx concentration between SF and uninjured recruits at any stage of training.

Thus, differences in anthropometric, aerobic fitness, baseline serum 25(OH)D concentration, bone mass and bone structure, between SF and uninjured recruits, provided evidence to inform changes in RM physical selection standards to reduce injury. Further work examining the effects of vitamin D supplementation on SF risk has been initiated to support recruit health and wellbeing.

